# The Role of Circulating Regulatory T Cell Levels on Subclinical Atherosclerosis and Cardiovascular Risk Factors in Women with Systemic Lupus Erythematosus

**DOI:** 10.1155/2018/3271572

**Published:** 2018-12-18

**Authors:** Mario García-Carrasco, Pamela Soto-Santillán, Claudia Mendoza-Pinto, Rebeca González-Ramírez, Ana Lidia López-Carmona, Pamela Munguía-Realpozo, Ivet Etchegaray-Morales, Socorro Méndez-Martínez, José Luis Gálvez-Romero, Aurelio López-Colombo, Alejandro Ruiz-Arguelles

**Affiliations:** ^1^Systemic Autoimmune Diseases Research Unit, Medical Unit of High Specialty, Manuel Ávila Camacho National Medical Center-CIBIOR, Instituto Mexicano del Seguro Social, Puebla, Mexico; ^2^Immunology and Rheumatology, Medicine School, Benemérita Universidad Autónoma de Puebla, Mexico; ^3^Medical Sciences and Research, Medicine School, Benemérita Universidad Autónoma de Puebla, Mexico; ^4^Laboratorios Clínicos de Puebla, Puebla, Mexico; ^5^Coordination in Health Research, Puebla Delegation, Instituto Mexicano del Seguro Social, Puebla, Mexico; ^6^Immunology Department, Instituto del Seguro Social al Servicio de los Trabajadores del Estado, Puebla, Mexico

## Abstract

The increase in cardiovascular disease (CVD) in patients with systemic lupus erythematosus (SLE) is not fully explained by traditional CVD risk factors. Regulatory T cells (Treg cells) are considered atheroprotective. We investigated the relationship between the absolute number of different phenotypes of Treg cells and abnormal carotid intima-media thickness (IMT) in women with SLE. Sixty-six women with SLE with no history of CV disease were included. Carotid IMT was quantified by ultrasound. Abnormal carotid IMT was defined as ≥0.8 mm and two groups were compared according to this definition. Flow cytometry was used to analyze Foxp3 and Helios expression in peripheral blood CD4 T cells. A significantly higher level of absolute CD4+CD25+FoxP3^high^ T cells was present in patients with abnormal carotid IMT compared with those without (1.795 ± 4.182 cells/*μ*l vs. 0.274 ± 0.784 cells/*μ*l; *p* = 0.003). However, no correlations were found between any Treg cell phenotypes and carotid IMT. Only the absolute number of CD4+CD45RA+FoxP3^low^ T cells was significantly decreased in SLE patients with low HDL cholesterol compared with those with normal HDL cholesterol (0.609 ± 2.362 cells/*μ*l vs. 1.802 ± 4.647 cells/*μ*l; *p* = 0.009 and 15.358 ± 11.608 cells/*μ*l vs. 28.274 ± 34.139; *p* = 0.012, respectively). In conclusion, in SLE women, diminished levels of Treg cells based on flow cytometry were not a good indicator of abnormal carotid IMT.

## 1. Introduction

In systemic lupus erythematosus (SLE) patients, cardiovascular disease (CVD) caused by atherosclerosis is more frequent than in the general population [1–4]. Conventional cardiovascular (CV) risk factors cannot explain this increased risk [2]. Accelerated atherosclerosis has been related to longer disease duration, supporting the assumption that chronic SLE immune dysregulation provokes atherosclerosis. Systemic inflammation, dysregulated cytokine profile, and altered T cell subsets have been proposed as important role players in endothelial dysfunction and increased CV risk in SLE patients [5]. Therefore, identification of CV damage and repair biomarkers related to SLE may reveal insights into the disease pathogenesis and might be used to monitor the CV risk and improve CV health.

With respect to the T cell subsets, the greatest number of pathogenic cells in the atherosclerotic process belongs to the T helper (Th) 1 profile, generating proinflammatory mediators such as interferon gamma (IFN-*γ*). The role of Th2 cells in atherogenesis remains unclear and the role of Th17 is under investigation [6]. Recently, a subset of specialized T cells with anti-inflammatory attributes, the regulatory T cells (Treg cells), which play a crucial role in the control of inflammation and autoimmunity, including chronic vascular inflammation causing atherosclerosis, has been studied. An increase in Treg cells was shown to correlate with a reduction in atherosclerosis in animal models [7], and Treg depletion promoted atherosclerosis in mouse models [8]. Treg cells might therefore be candidates for therapeutic interventions [9].

Defects in Treg cells might be an important issue in SLE pathogenesis. Nevertheless, reports on the number and function of Treg cells in SLE are contradictory [10], which may be due to the use of different markers to characterize Treg cells. An impaired number of CD4+CD25+FoxP3+ Treg cells have been found in SLE patients [11]. Moreover, CD4+CD25+FoxP3+ T cells positively correlated with disease activity [12]. It has been reported that some FoxP3+ cells are phenotypically naive (CD45RA+) in peripheral blood and have a suppressive function [13]. Miyara et al. found that CD45RA+FoxP3^low^ Treg cells which are suppressive in vitro [14] are increased active SLE and Pan et al. found that CD45RA+FoxP3^low^ T cells contribute to SLE progression with aberrant inflammatory status [15]. Studies have also analyzed new markers for Treg cells with regard to SLE, for example, Helios, which is a member of the Ikaros gene family of transcription factors. Helios is preferentially expressed in mRNA and proteins in Treg compared with naive T cells [16]. High percentages of FoxP3+Helios+ cells have been found within the circulating CD4+ population in active SLE, and the numbers of FoxP3+Helios+ cells have been shown to correlate with disease activity [17, 18]. However, few studies have focused on the role of Treg cells in atherosclerosis in SLE patients [19, 20]. Moreover, given the role of T cells in atherosclerosis, they might be an important target to consider when developing therapies to treat SLE and CVD. The aim of this study was to compare the number of Treg cells in SLE patients with and without subclinical atherosclerosis.

## 2. Materials and Methods

### 2.1. Study Population

From August 2015 to February 2017, 66 consecutive mixed-race SLE patients were recruited at the Systemic Autoimmune Diseases Research Unit outpatient clinic (Hospital General Regional 36, IMSS). All fulfilled ≥4 American College of Rheumatology criteria for SLE [21]. Inclusion criteria were age ≥ 18 and <65 years and no history of overt CV events. Exclusion criteria were previous cardiovascular events (myocardial infarction, angina, arrhythmia, congestive heart failure, stroke, or peripheral arterial disease), smoking (current or within the previous 6 months), overweight (body mass index ≥ 30 kg/m^2^), current pregnancy, and current infection. The study was approved by the local ethics committee. Study participants gave written informed consent.

### 2.2. Clinical Assessments

Traditional CV risk factors age, sex, family history of CVD, body mass index, waist circumference, hypertension, and dyslipidemia were assessed. Because the traditional Framingham risk factor score underestimates the risk for coronary artery disease (CAD) in patients with SLE, we calculated a modified Framingham risk score by multiplying the items by two [22]. High waist circumference (≥102 cm for men and ≥88 cm for women), hypertriglyceridemia (≥150 mg/dl or 1·7 mmol/l or specific treatment for this lipid abnormality), elevated blood pressure (systolic ≥ 130 mmHg or diastolic ≥ 85 mmHg or taking medication for hypertension), and elevated fasting plasma glucose (≥110 mg/dl or 6.1 mmol/l) or a diagnosis of type 2 diabetes were recorded.

SLE-specific measures: SLE activity was measured using the SLE Activity Disease Activity Index (SLEDAI-2K) [23] and accumulated damage by the Systemic Lupus International Collaborating Clinics/ACR (SLICC/ACR) Damage Index [24]. Medication, including statins, antiplatelet, antimalarials, prednisone daily dose, cumulative glucocorticoid dose, and immunosuppressive treatments, was recorded. The diagnosis of antiphospholipid syndrome (APS) was based on a history of venous and/or arterial thrombosis or recurrent miscarriages with positive antiphospholipid antibodies according to published criteria [25]. Blood tests included complete blood cell count, creatinine, total cholesterol, high-density lipoprotein cholesterol (HDL-C), low-density lipoprotein cholesterol (LDL-C), triglycerides, anti-DNA antibodies, and complement.

### 2.3. Enumeration of Treg Cells

Treg lymphocytes were detected in peripheral blood samples obtained in EDTA_K3_-containing tubes. Briefly, 50 microliters of blood was stained for 15 minutes with CD4-Alexa Fluor 700, CD25-APC, and CD45RA-PE (Beckman Coulter) fluorescent monoclonal antibodies and incubated for 10 minutes at room temperature. After the addition of 50 *μ*l of a fixative formaldehyde solution (IntraPrep® Reagent 1), stained cells were incubated for 15 minutes and washed with isotonic buffered saline solution by centrifugation. Cells were then exposed simultaneously to a permeability agent consisting of saponine (IntraPrep® Reagent 2) and monoclonal antibodies to FoxP3 and Helios A488 (FITC) and incubated for 15 more minutes. Finally, cells were washed and resuspended in isotonic buffered saline for analysis (monoclonal antibodies to Helios, FoxP3, and CD45RA were purchased from Becton Dickinson, whilst CD45, CD25, CD4, and the IntraPrep Kit were obtained from Beckman Coulter).

Stained cells were analyzed by flow cytometry (Galios, B5 R1 V2 configuration, Beckman Coulter Inc., Fullerton, CA, USA). Lymphocytes were gated with CD45 and side scatter criteria, and CD4-positive cells were further gated from lymphocytes. CD4+/FoxP3+, CD4+/Helios+, and CD4+/CD45RA subpopulations were enumerated by Boolean gating in CD4+.

A minimum of 20,000 lymphocytes was collected to enumerate the absolute number [26] of cell subpopulations. Absolute number of T cells was calculated using a double platform method, i.e., the relative values obtained from the flow cytometry analysis were converted to the total number of cells/ml of blood using the following calculation: Absolute (Abs) CD4 cells = %CD4 cells × total WBC count divided by 1000 (expressed as a decimal): this enables the absolute count per mcl of each cell type to be determined. The absolute WBC count was derived using a Coulter DxH 600 automated hematology analyzer.

Specifically, four phenotypically different subsets were evaluated on the basis of the expression of CD45RA, FoxP3, CD217, and Helios, which have been found in SLE patients: (1) CD4+CD25+FoxP3^high^ [11, 12], (2) CD4+CD45RA+FoxP3^low^ [15], and (3) CD4+FoxP3+Helios+ [17, 18].

### 2.4. Carotid Ultrasound

Carotid intima-media thickness (IMT) was assessed by B-mode ultrasonography. One centimeter segments of bilateral common carotid arteries, carotid bulbs, and distal internal carotid arteries were scanned. Images were obtained of the near or far wall of each arterial segment per standardized procedures [27]. The standard ultrasound machine used was the Philips iU22 (C5-2 Probe, iU22 Intelligent Ultrasound System; Philips, Bothell, WA, USA). Abnormal carotid IMT was defined as IMT ≥ 0.8 mm which is beyond the 75th percentile as per the American Society of Echography Task Force recommendations [28]. Plaque was defined as a protrusion into the vessel lumen of ≥1.5 mm, as measured from the border between the adventitial and medial layer. The percentage of vessel obstruction was measured along the longitudinal axis and classified as stenosis < 20%, from 20 to 49%, from 50 to 69%, and from 70 to 99% [18].

### 2.5. Statistical Analysis

Descriptive statistics were calculated for categorical variables as proportion (%) and continuous variables as mean ± standard deviation or median (interquartile range, IQR) if the distribution was skewed. To compare baseline characteristics between patients with and without abnormal IMT, we used the chi-square test for categorical variables and the Student *t*-test for continuous variables. Bivariate correlations were analyzed using Spearman's rank correlation coefficient. A probability value < 0.05 was considered significant. All calculations were made using SPSS software (Version 22.0 for Mac; SPSS Inc., Chicago, IL, USA).

## 3. Results

### 3.1. Participant Characteristics

The sociodemographic and clinical characteristics of the SLE patients included are shown in Table 1. Six (9.1%) patients had APS, of which only one patient had triple antiphospholipid antibody positivity. Most of the patients had mild lupus activity at the moment of the study. Therapies for SLE patients were prednisone in 62 patients (93.3%, mean dose 12.29 ± 9.75 mg/day); median (IQR) of cumulative glucocorticoid (GCT) dose was 24.50 (9.25–38.6); antimalarials in 49 (74.2%); immunosuppressive agents in 39 (59.1%). Eleven patients (16.6%) were on a statin. The median (IQR) Framingham risk score for CV events occurring within the next 10 years was 1% (<1-2%).

### 3.2. Between-Group Differences in Sociodemographic and Clinical Characteristics

Thirty-nine (59.0%) patients had an abnormal carotid IMT. Sociodemographic, clinical, and therapy variables in patients with and without abnormal carotid IMT are shown in Table 2. Only APS was slightly but significantly more frequent in SLE patients without abnormal carotid IMT. There were no significant differences in age, menopause status, smoking history, and HDL-C or LDL-C values between groups. The positive rate of anti-ds-DNA did not differ significantly between groups. Although patients with abnormal carotid IMT had higher triglyceride levels, the difference was not significant.

### 3.3. Correlation between Circulating Regulatory T Cells, Cardiovascular Risk Factors, and Pharmacological Treatment

The absolute number of CD4+ T cells was significantly higher in the group of SLE patients with abnormal carotid IMT than SLE patients with normal carotid IMT (Table 1). The absolute number of CD4+CD25+FoxP3^high^ T cells was greater in patients with hypertriglyceridemia and elevated blood pressure compared with patients without these conditions (1.654 ± 4.380 cells/*μ*l vs. 0.628 ± 1.265 cells/*μ*l; *p* = 0.01 and 2.445 ± 7.195 cells/*μ*l vs. 0.945 ± 2.056 cells/*μ*l; *p* = 0.03, respectively). In contrast, the absolute number of CD4+FoxP3+Helios+ T cells did not differ significantly between patients with and without these conditions. However, higher levels of CD4+CD45RA+FoxP3^low^ T cells were found in patients with hypertriglyceridemia compared with those without (1.677 ± 4.543 cells/*μ*l vs. 0.693 ± 2.496 cells/*μ*l; *p* = 0.03).

The mean absolute number of CD4+CD25+FoxP3^high^ T cells was higher in patients with high waist circumference (≥88 cm for women) than in those with normal waist circumference (1.832 ± 4.593 cells/*μ*l vs. 0.180 ± 0.326; *p* = 0.007). The absolute number of CD4+CD45RA+FoxP3^low^ T cells was significantly lower in patients with low HDL cholesterol than in those without (0.609 ± 2.362 cells/*μ*l vs. 1.802 ± 4.647 cells/*μ*l; *p* = 0.009 and 15.358 ± 11.608 cells/*μ*l vs. 28.274 ± 34.139; *p* = 0.012, respectively). No correlations were observed between any absolute number of Treg cells and the Framingham risk score. No significant differences in Treg cell numbers were found in patients under therapy with aspirin or statins.

### 3.4. Between-Group Differences in Regulatory T Cells in Patients with and without Abnormal Carotid IMT

**Figure 1:**
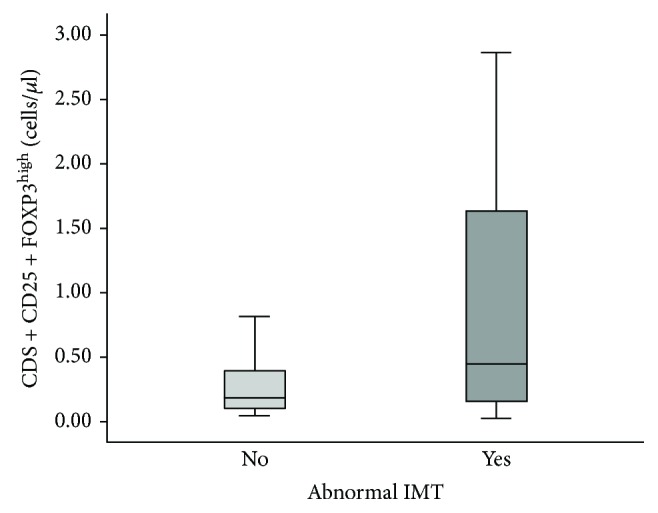
Box plot of absolute number of CD4+CD25+FOXP3^high^ T cells in SLE women with and without abnormal carotid IMT.

Table 3 compares Treg cell phenotypes between patients with and without abnormal carotid IMT. Although the absolute number of CD4+CD25+FoxP3^high^ T cells was significantly higher in patients with abnormal carotid IMT compared with those without abnormal carotid IMT (Figure 1), no correlations were found between any Treg cell phenotype and carotid IMT. Other Treg cell phenotypes (CD4+CD45RA+FoxP3^low^ and CD4+FoxP3+Helios+) were not correlated with carotid IMT.

## 4. Discussion

Studies have suggested an important role of Treg cells in CV disease. Animal models consistently show that an increase in Treg cell levels and function is associated with a decreased risk of atherosclerosis [7, 8]. However, results from human studies are unclear. We investigated Treg cell levels based on flow cytometry analysis and studied their relationship to subclinical atherosclerosis in SLE patients. We found that levels of circulating CD4+CD25+FoxP3^high^ cells tended to be higher in patients with an increased carotid IMT. However, all phenotypes evaluated in our study (CD4+CD25+FoxP3^high^, CD4+CD45RA+FoxP3^low^, and CD4+FoxP3+Helios+ cells) were not significantly correlated with abnormal carotid IMT in SLE women with low disease activity and a low risk of CV. These findings support and extend the observations from other studies [29] evaluating circulating CD4+CD25^high^CD127^low^ regulatory T cell levels. A possible explanation could be that in humans circulating Treg-cell levels are not directly related to the extent of atherosclerosis. Our observations do not exclude the possibility that local variations in Treg-cell levels/function affect vascular lesion progression to atherosclerosis and do not rule out the possibility that circulating Treg cell levels are altered and play a role in clinical atherosclerosis.

Treg cells have been associated with certain CV risk factors, such as hypertension, increased serum cholesterol, and obesity [30, 31]. In our study, the Framingham risk score did not correlate with any of the Treg cell phenotypes investigated; however, some Treg cell phenotypes differed significantly between patients with and without hypertriglyceridemia, low HDL, elevated blood pressure, and high waist circumference. This suggests that some subjects with increased inflammatory status may also have increased circulating Treg cell levels. In contrast, a significant inverse correlation between levels of HDL cholesterol and circulating CD4+CD25^high^CD127^low^ has been observed [29]. Recently, it was shown that the frequency of CD4+FOXP3+ T cells correlated positively with HDL cholesterol levels in healthy adults treated with statins [32]. However, we found no differences in Treg cells in patients with and without statin therapy, in contrast to other studies. This may be because our study was not powered to find differences between patients using statins [33].

The role of the adaptive immune response in atherosclerosis is increasingly recognized. It is accepted that atherosclerosis is caused by an imbalance between pathogenic T cells and Treg cells. In the atherosclerotic inflammatory process, the role of Treg cells could have a suppressive function, preventing the accumulation of proinflammatory cells and the secretion of proinflammatory cytokines [34]. The direct role of Treg cells in experimental atherosclerosis models initially described depletion of peripheral Treg cells and increased atherosclerotic lesion size and its vulnerability in animal models [35]. In addition, some studies have found fewer naturally occurring Treg cells with impaired suppressive properties in patients with acute coronary syndrome who have coronary atherosclerosis [36]. In SLE patients, one study found an imbalance, with a dominance of Th17 over Treg cells, a finding also implicated in SLE-related atherosclerosis [37]. Moreover, a recent study found that IgM antibodies against phosphorylcholine promote polarization of Treg cells in SLE patients with atherosclerotic plaques, which could represent a novel protective mechanism in atherosclerosis and autoimmune conditions, such as SLE [38].

We found that circulating Treg cell levels were not associated with carotid IMT in SLE women at low risk for CV events. These findings are consonant with the observations from two small studies [39, 40]. Recently, a study of the value of carotid IMT in predicting CV risk reported that although IMT is associated with myocardial infarction and stroke [41], plaque area seems to be more representative of the atherosclerotic process [42]. However, among our patients included in the carotid study, only one patient had carotid plaque, and therefore, we were not able to evaluate the role of Treg cells in this condition in our patients.

New markers for the characterization of Treg cells, such as CD4+FoxP3+Helios+, have been analyzed. A number of correlative experiments suggested that FoxP3+Helios+ Treg cells were thymus-derived. Although some studies in mice have demonstrated that Helios can be expressed on induced FoxP3+ T cells, there is little doubt that Helios expression is a valid marker of functional Treg cells. It has been suggested that the combination of Helios and FoxP3 is a superior method for identifying Treg cells in SLE patients [17, 18]. Our study found no correlation between this Treg cell phenotype and carotid IMT or any cardiovascular risk factor evaluated. This may be explained, in part, by the fact that our patients had low disease activity, since this phenotype has been shown to be present in high percentages in active SLE patients [17, 18]. Moreover, the role of Helios expression in Treg cells in atherosclerosis is little studied. Recently, a Chinese study demonstrated that circulating Helios+Tregs are significantly decreased in acute coronary syndrome and the estimated absolute numbers of CD4+Foxp3+Helios+ Tregs were negatively correlated with IL-6 and positively correlated with circulating levels of the transforming growth factor beta1 (TGF-beta1) and HDL-C in these patients [43].

The study had some limitations. Firstly, the cross-sectional design and small sample size may limit the ability to infer a causal relationship between Treg cells and subclinical atherosclerosis. Secondly, although we evaluated several Treg cell phenotypes shown to be present in SLE patients, other phenotypes, such as CD4+CXCR5+FoxP3+ [44] and CD4+CD25^low/-^GITR+ cells [45], were not evaluated. Thirdly, functional profiles to determine the regulatory activity of the Treg cell phenotypes included were not made. Fourthly, new atherosclerosis imaging markers explored in SLE, including computerized tomography angiography [46] and flow-mediated dilation [47], were not used. Finally, carotid plaque was present in only one patient, and therefore, we could not compare levels of Treg cell phenotypes between patients with and without carotid plaques. Studies have shown that subjects with stable plaque evaluated by coronary angiography had significantly higher levels of CD4+CD25+Foxp3+ cells compared with patients with vulnerable plaque.

In conclusion, studies that reproducibly evaluate circulating Treg cells in SLE patients are fundamental to define Treg cell reference ranges and the importance of their variability in patients with subclinical manifestations of atherosclerosis. We found no significant correlations between circulating Treg cell numbers, identified by CD4+CD25+FoxP3^high^, CD4+CD45RA+FoxP3^low^, and CD4+FoxP3+Helios+ assessments and carotid IMT in SLE women with low disease activity and at low risk of CV events. The reason may lie in the complexity of defining Treg cells in SLE patients, in whom the analysis of activated T cells is challenging.

## Figures and Tables

**Table 1:** Baseline characteristics of SLE patients (*n* = 66).

Variable	SLE (*n* = 66)
Age (years), mean ± SD	37.81 ± 8.33
Framingham score median (IQR)	1.40 (1.40, 2.00%)
BMI (kg/m^2^), mean ± SD	25.61 ± 2.98
Systolic BP (mmHg), mean ± SD	111.55 ± 15.22
Diastolic BP (mmHg), mean ± SD	69.78 ± 10.31
Total cholesterol (mg/dl), mean ± SD	195.18 ± 36.11
Triglycerides (mg/dl), mean ± SD	173.33 ± 87.51
High-density lipoproteins (mg/dl), mean ± SD	49.86 ± 15.17
Low-density lipoproteins (mg/dl), mean ± SD	109.71 ± 29.08
Family history of CVD, *n* (%)	15 (22.7%)
*SLE-specific measures*	
Disease duration (years), mean ± SD	10.64 ± 4.88
Antiphospholipid syndrome, *n* (%)	6 (9.1)
SLEDAI-2K score, median (IQR)	1.0 (0.0, 4.0)
SLICC damage index, median (IQR)	0.0 (0.0, 1.0)
SLE manifestations, *n* (%)	
Mucocutaneous manifestations	43 (65.1)
Articular involvement	41 (62.1)
Renal involvement	21 (31.8)
Hematological involvement	25 (37.8)
Serositis	8 (12.1)
Neuropsychiatric manifestations	0 (0)
Elevated anti-dsDNA antibody^†^, *n* (%)	10 (15.2)
Complement C3 (mg/l), mean ± SD	103.9 ± 28.3
Complement C4 (mg/l), mean ± SD	39.4 ± 17.9
*CVD surrogates*	
Carotid IMT (mm), mean ± SD	0.85 ± 0.21

BMI: body mass index; BP: blood pressure; CVD: cardiovascular disease; dsDNA: double-stranded DNA; IMT: intima-media thickness; IQR: interquartile range; SD: standard deviation; SLEDAI-2K: SLE Disease Activity Index 2000; SLICC: Systemic Lupus International Collaborating Clinics. ^†^As per laboratory reference range.

**Table 2:** Clinical characteristics and biological parameters in patients with and without abnormal IMT.

Characteristic	Abnormal carotid IMT (*n* = 39)	Normal carotid IMT (*n* = 27)	*p* value
Age (years), mean ± SD	37.59 ± 8.14	38.16 ± 8.77	0.71
Menopause, *n* (%)	10 (25.64)	5 (18.51)	0.39
Smoking, *n* (%)	3 (7.69)	0 (0.0)	0.23
Diabetes, *n* (%)	0 (0)	2 (7.40)	0.14
Hypertension, *n* (%)	8 (20.5)	2 (7.4)	0.13
BMI (kg/m^2^), mean ± SD	25.68 ± 2.95	25.50 ± 3.09	0.93
Waist circumference, mean ± SD	89.65 ± 7.86	86.77 ± 9.48	0.29
Systolic BP (mm Hg), mean ± SD	112.97 ± 17.25	109.42 ± 11.51	0.09
Diastolic BP (mm Hg), mean ± SD	70.64 ± 10.83	68.50 ± 9.55	0.76
Total cholesterol, mean ± SD	198.48 ± 36.06	190.41 ± 40.28	0.27
LDL cholesterol, mean ± SD	111.06 ± 27.10	107.76 ± 32.2	0.57
HDL cholesterol, mean ± SD	50.05 ± 14.06	49.56 ± 17.07	0.50
Triglycerides, mean ± SD	179.56 ± 91.90	164.33 ± 81.61	0.55
10-year risk of heart attack (%), mean ± SD	1.73 ± 0.70	1.60 ± 0.54	0.24
Duration of SLE disease (years), median (IQR)	10 (7. 11)	10 (7. 13)	0.98
SLEDAI-2K score, median (IQR)	1 (0. 3)	1 (1. 4)	0.50
Antiphospholipid syndrome, *n* (%)	**1 (2.6)**	**5 (18.5)**	**0.03**
Prednisone daily dose, mg, mean ± SD	12.67 ± 10.83	11.73 ± 8.08	0.39
Cumulative dose of steroid treatment (g), median (IQR)	22.05 (9.75. 36.00)	24.95 (8.32. 37.72)	0.13
Antimalarials, *n* (%)	29 (74.35)	20 (74.07)	0.60
Immunosuppressive drugs, *n* (%)	24 (61.53)	15 (55.55)	0.32
Statin therapy, *n* (%)	11 (28.20)	9 (33.33)	0.42
Antiplatelet therapy, *n* (%)	11 (28.20)	9 (33.33)	0.36
Vitamin K antagonist therapy, *n*	1 (2.5)	4 (14.8)	0.11
Antihypertensive therapy, *n* (%)	10 (25.6)	7 (25.9)	0.86

BMI: body mass index; BP: blood pressure; HDL: high-density lipoprotein; LDL: low-density lipoprotein; SD: standard deviation; SLEDAI-2K: SLE Disease Activity Index 2000.

**Table 3:** Frequency and phenotype of peripheral blood Tregs in SLE patients with and without abnormal carotid IMT determined by flow cytometry.

Markers for Treg detection	Abnormal carotid IMT (*n* = 39)	Normal carotid IMT (*n* = 27)	*p* value
CD4+ (cells/*μ*l)	534.565 ± 265.858	377.576 ± 125.343	**<0.001**
CD4+CD25+FoxP3^high^ (cells/*μ*l)	1.795 ± 4.182	0.274 ± 0.784	**0.003**
CD4+CD45RA+FoxP3^low^ (cells/*μ*l)	2.032 ± 4.703	0.034 ± 0.119	0.067
CD4+FoxP3+Helios+ (cells/*μ*l)	0.020 ± 0.064	0.037 ± 0.154	0.306

Data are expressed as mean ± standard deviation; the absolute number of cells is expressed as cells/*μ*l unit.

## Data Availability

The data used to support the findings of this study are available from the corresponding author upon request.
